# The relationship between epicardial fat thickness and high-grade varicocele

**DOI:** 10.4314/ahs.v20i4.28

**Published:** 2020-12

**Authors:** Isa Sincer, Emrah Erdal, Adnan Gucuk, Emre Bostancı, Yilmaz Gunes

**Affiliations:** 1 Abant Izzet Baysal University, Department of cardiology; 2 Abant Izzet Baysal University, Department of Urology

**Keywords:** High-grade varicocele, epicardial fat thickness, flow-mediated dilatation, aortic stiffness

## Abstract

**Background:**

Varicocele is abnormal dilation of testis veins. The precise mechanism of varicocele is not fully understood despite some hypothesis were suggested in the literature. Disequilibrium between constrictor and dilatator mechanism of the veins have been shown to cause varicocele. High-grade varicoceles have been also linked to endothelial dysfunction and increased vasoconstriction.

**Objectives:**

We hypothesized that epicardial fat thickness (EFT), flow-mediated dilatation (FMD) and aortic stiffness (AS) could be associated with varicocele. In the present study, we aimed to compare vascular parameters such as FMD, EFT and AS in healthy subjects and high-grade varicocele patients.

**Methods:**

The study population consisted of 35 men with high-grade varicocele and 32 age- and sex-matched control subjects younger than 45 years old. This is a cross-sectional study conducted at Bolu Abant Izzet Baysal University Hospital between May to October 2018.

**Results:**

EFT, aortic diastolic diameters (AoDD) and EFT/BMI ratio were significantly higher in control group than in patients with high-grade (p=0.012, p=0.044, p=0.026, respectively). EFT and EFT /BMI ratio were significantly and inversely correlated with presence of varicocele (r=-0.422, p=0.009; r=-0.38, p=0.026, respectively).

**Conclusion:**

The present study suggests that high-grade varicocele may be associated with decreased echocardiographic EFT but not with aortic stiffness and FMD.

## Introduction

Varicocele is an abnormal dilation of testis veins with an incidence of 10% to 15% of children and adolescents and in 40% of the males with infertility^1^,^2^. The precise mechanism of varicocele is not fully understood but some hypotheses were suggested in the literature. Disequilibrium between constriction and dilatation mechanisms of the veins have been shown to cause varicocele. High-grade varicoceles have been also linked to endothelial dysfunction and increased vasoconstriction^3^. Moreover, the co-existence of arterial and venous conditions, such as varicosities of the coronary venous system and the leg veins, have been demonstrated previously^4^. The goal of varicocele treatment is to eliminate the dilatation of the pampiniform venous plexus and, hence, to obstruct the refluxing venous drainage to the testis^5^. Flow-mediated dilation (FMD) is a marker of endothelial function. It is measured by the capacity of the brachial artery to dilate in response to ischemia induced by hyperemia and reflects the local bioavailability of nitric oxide (NO) under a physiologic stimulation. Aortic stiffness (AS) is a parameter of the biomechanical properties of the aorta, which reflects aortic distensibility and elasticity, and mechanical strength to the aortic wall. Changes in aortic stiffness and FMD have been associated with various inflammatory diseases such as rheumatoid arthritis, systemic vasculitis, systemic lupus erythematous, inflammatory bowel disease, and coronary artery disease^6^. These findings suggest that varicocele may be a consequence of systemic disease associated with diffuse vascular abnormalities. Since inflammation plays an important role in both coronary artery ectasia and peripheral varicose veins, it can be expected that an indirect correlation may exist between these two conditions^7^,^8^. As a white adipose tissue (WAT), pericardial fat, which covers about the 80% of heart's surface, makes the 20% of the total weight of the heart. It may act as an endocrinologically active tissue via paracrine and vasocrine mechanisms and regulates the functions of the heart and blood vessels. Epicardial fat thickness (EFT) has been reported to be associated with several conditions such as coronary artery disease (CAD), metabolic syndrome, right ventricular systolic dysfunction (RVSD), inflammatory bowel disease (IBD) and left ventricular dysfunction^9^–^12^. We hypothesized that EFT, FMD and AS could be associated with varicocele. For this purpose, in the present study, we aimed to compare vascular parameters such as FMD, EFT and aortic stiffness in healthy subjects and highgrade varicocele patients.

## Methods

The study population consisted of 35 men with grade 3 varicocele and 32 age matched control subjects younger than 45 years old, without previous testicular surgery. Patients with coronary artery disease (CAD), left ventricular systolic heart failure (EF< 50%), moderate to severe mitral and/or aortic valvular heart disease, congenital heart disease, atrio-ventricular conduction abnormalities, pericardial effusions, moderate to severe renal or liver disease, thyroid disorders, anemia, diabetes mellitus, dyslipidemia, hypertension, conditions that associated with electrolyte imbalances, any systemic inflammatory or infectious disease and inadequate transthoracic echocardiographic imaging were excluded. For 85% power according to t test, alpha value used was 0.08, the sample size was calculated as 60. This was a cross-sectional study conducted at Bolu Abant Izzet Baysal University Hospital between May to October 2018. We obtained informed consent from each participant after approval of the study protocol by the local Ethics Committee.

Scrotal color Doppler ultrasound was used to define high-grade varicocele (Grade 3) as following: Grade 0: normal diameter of Pampiniform plexus (<2 mm ); Grade 1:Mild dilatation of the pampiniform plexus (2–3 mm); Grade 2: Moderate dilatation (3–5 mm); Grade 3: Longer than 1 second reflux following Valsalva maneuver which is independent of vessel diameter^13^.

4-Mhz transducer of Vivid S6 (GE Vingmed, N-3191 Horten-Norvay) was used in all echocardiographic examinations. A cardiologist blinded to the study performed the echocardiological assessments. One-lead electrocardiography (ECG) was recorded continuously, and three consecutive cycles were averaged for every measured parameter during echocardiographic examination. Echocardiographic examinations were performed with patients in the left lateral position by a single operator. The following measurements were obtained by: left ventricular end-diastolic diameter (LVEDD, mm), left ventricular end-systolic diameter (LVESD, mm), interventricular septum thickness, left ventricular posterior wall thickness, left ventricular ejection fraction (LVEF %) and EFT. Simpson's rule was used in prediction of LVEF. The method defined by Lacobelli was used to measure EFT^14^. The echo-free space between the outer wall of the myocardium and the visceral layer of the pericardium was identified as epicardial fat and it was calculated at the end-diastole in three cardiac cycles perpendicularly on the free wall of the right ventricle. The average of the maximum value measured at any site was used. Body mass index (BMI) was calculated by division of weight in kilograms by the square of height in meters. The EFT to BMI ratio (EFT/BMI) values were measured by division of EFT by BMI count.

Internal dimensions of the ascending aorta were measured in at least three consecutive cardiac cycles. The measurements were carried out in the proximal ascending aorta, 3 cm from the origin of the aorta. At the maximal anterior motion of the aorta and at the beginning of the QRS complex on the simultaneously recorded electrocardiogram. We measured the aortic systolic diameters (AoSD) and the diastolic diameters (AoDD), respectively. Aortic strain, aortic distensibility, and aortic stiffness index were calculated using the following formulas^15^.

1. Aortic diameter change (ADC): AoSD – AoDD
2. Aortic strain (AS) (%): ADC/AoDD
3. Aortic distensibility (cm2 dyn – 1 10 – 3): 2xAS/PP
4. Aortic stiffness index: (SBP/DBP)/[(ADC)/AoDD].

FMD, aortic stiffness and EFT were primary outcome measures of present study. Before FMD measurement subjects were instructed to fast for at least 8 hours before undergoing FMD and to abstain from exercising, smoking, ingesting alcohol and caffeine, all of which may affect FMD measurements. All subjects rested for at least 15 min in a supine position in a quiet, dark, air-conditioned room (22–25 °C). The brachial artery diameter was measured in the antecubital fossa just before giving its branches using a Doppler ultrasound system with a high resolution 7.5 MHz linear array transducer (GE healt care, M4S-RS, Tokyo, Hinoshi, Japan). When a satisfactory transducer position with clear anterior and posterior intimal interfaces between the lumen and vessel wall was found, the surface of the skin was marked and all measurements were obtained from the same area. After acquiring a baseline rest image, a sphygmomanometric (blood pressure) cuff was placed and inflated to 250 mmHg (or at least 50 mmHg above systolic pressure) to occlude inflow in the brachial artery for 5 min. Reactive hyperemia was created in the brachial artery after sudden cuff released. Percent-change in FMD (%) was calculated as the percent change in the vessel diameter between the baseline value and maximum dilatation during reactive hyperemia (FMD=100 x (maximum diameter after hyperemia-baseline diameter)/baseline diameter).

Analyses were carried out using SPSS 18.0 Statistical Package Program for Windows (SPSS Inc, Chicago, Illinois, USA). Normally distributed quantitative variables were expressed as mean ± standard deviation (SD) and as median in case of non-normal distribution. Qualitative variables were expressed as numbers and percentages. Differences between independent groups were assessed by the Student t-test for normally distributed quantitative variables and the Mann-Whitney U-test for variables without normal distribution and the Chi-square test for qualitative variables. Pearson correlation analyses was used to assess the correlations between EFT, EFT/BM and varicocele grade. A receiver operating curve (ROC) analysis was used to find sensitivity and specificity of EFT to predict the presence of varicocele. All results were considered statistically significant at a level of p <0.05.

## Results

The study population included 67 subjects: 32 in control group and 35 in grade 3 varicocele group. Baseline patient demographics, including age and clinical risk factors, were similar between the groups. Although not significant, the SBP tended to be higher in varicocele group (Table-1).

**Table 1:** Baseline characteristics of the study population

Baseline characteristics	Control (n=32)	Varicocele(n=35)	p
	**Median (IQR)**		
Age (years)	27(6)	26 (8)	0.612
SBP (mmHg)	118 (20)	120 (10)	0.057
DBP (mmHg)	75 (10)	76 (11)	0.866
	**Mean ±standard** **deviation**		
Body mass index (kg/m2)	25±3	25±4	0.843
Smoking	10(31%)	12(34%)	0.142

DBP: diastolic blood pressure, SD: standard deviation, SBP: systolic blood pressure.

IOR, interquartile range

Echocardiographic parameters including LVEF, LVDD, LVSD, PW, IVSD, AoSD, FMD and ASI were similar between varicocele and control groups. However, median EFT of varicocele group (0.50 (0.10)) was significantly less than that of the control subjects (0.51 (0.12)) (p=0.012). Similarly, median AoDD of varicocele group (2.4 (0.4)) was shorter than control subjects (2.5 (0.5)) (p=0.044). EFT/BMI ratio of varicocele group (0.019 ±0.004) was also significantly lower than that of the controls (0.021±0.004) (p=0.026), (Table-2).

**Table 2:** Echocardiographic parameters and Doppler ultrasonographic data of the study cohorts

Variables	Control (n=32)	Varicocele(n=35)	p
	**Median (IQR)**		
EF	65(2)	64(5)	0.110
LVDD(cm)	4.6 (0.5)	4.7 (0.4)	0.267
LVSD(cm)	3.0 (0.6)	3.1 (0.5)	0.097
PW(cm)	0.9 (0.3)	0.9 (0.1)	0.116
IVST(cm)	0.8 (0.1)	0.8 (0.1)	0.804
AoSD(cm)	2.8 (0.3)	2.6 (0.3)	0.115
AoDD (cm)	2.5 (0.5)	2.4 (0.4)	0.044
ASI	2.5 (0.5)	2.4 (0.4)	0.115
Epicardial fat(cm)	0.51 (0.12)	0.50 (0.10)	**0.012**
	**Mean ±standard** **deviation**		
FMD	12.83±3.30	15.35±7.21	0.085
EFT to BMI ratio	0.021±0.004	0.019 ±0.004	**0.026**

AoDD: aortic diastolic diameter, ASI: aortic stiffness index, AoSD: aortic systolic diameter, EF: left ventricular ejection fraction, FMD: flow-mediated dilation, LVDD: left ventricular end-diastolic diameter, LVSD: left ventricular end-systolic diameter, PW: posterior wall thickness, IVST: interventriculer septum thickness, BMI: Body mass index, EFT: Epicardial fat thickness.

IOR: interquartile range

In Pearson's correlation test, EFT (r=-0.422, p=0.009) and EFT/BMI ratio (r=-0.38, p=0.026) were significantly and inversely correlated with presence of varicocele. An EFT level of < 0.505 cm had sensitivity, specificity, positive predictive value and negative predictive value of 52%, 82%, 64% and 73%, respectively (AUC = 0.678, 95% CI, 0.545–0.811) for the prediction of varicocele. Overall accuracy of EFT in determination of varicocele was 67% (Figure 1).

**Figure Legend-1:**
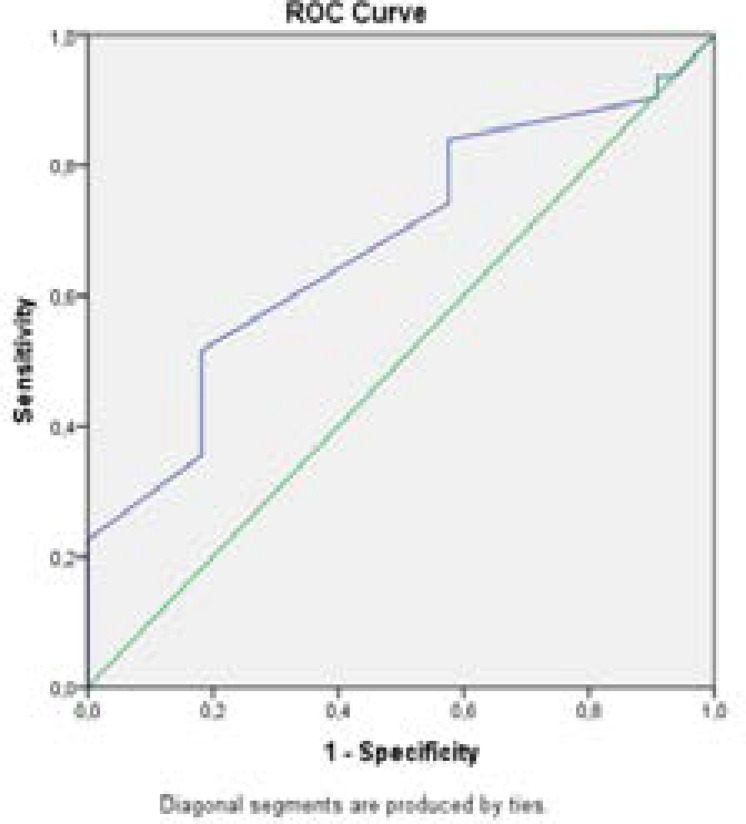
ROC curve analysis of EFT for prediction of varicocele. At the cut-off value of >0,505 cm, sensitivity and specificity of MPV were 52% and 82%, respectively (AUC = 0.678, 95% CI, 0.545–0.811). AUC: area under the curve, CI: Confidence interval.

## Discussion

In this study, we showed that EFT and EFT/BMI were significantly reduced in the varicocele group compared to the control group. While there was no correlation between BMI and varicocele group, both EFT and EFT/BMI were significantly and negatively correlated with varicocele. Another interesting result of the present study was that EFT greater than 0.505 cm had high specificity and negative predictive value in determining varicocele.

Besides its role as a lipid storage function, adipose tissue has also important role in regulating body energy, lipids, and carbohydrates metabolism^16^. Adipose tissue is simply grouped into two: white (WAT) and brown (BAT) adipose tissues. BAT is localized in the hypodermis and has great innervation and vascularization. In contrast, WAT is mainly localized in the internal organs and acts as a lipid and energy storage. EFT is a kind of WAT and localized between pericardial visceral surface and the myocardium. Since EFT provides cytokines and hormones to the bloodstream, it is accepted as an endocrine gland^17^. Furthermore, it has regulatory effect on heart and blood vessels by paracrine and vasocrine pathways. Thus, it is an active tissue and a great anti-inflammatory and proinflammatory cytokine source^17^.

EFT produces anti-inflammatory adipokines such as adinopectin, omentin and adrenomedullin. Adinopectin is secreted from adipose tissues and the adrenal gland. Adrenomedullin is secreted from the heart, lung, kidney and omentin from adipose tissues, especially the heart 18. These cytokine mediate regulatory effects of EFT are on the heart and vasculature^18^. Moreover, adrenomedullin, a strong vasodilatator in the blood stream, inhibits production of endotelin-1 and apoptosis of endothelial cells 18. Varicocele is a common cause of infertility in men with unclear etiology. Many studies in the literature pointed out a correlation between varicocele and coronary artery ectasia and peripheral venous insufficiency, 19. Inflammatory processes play a role in coronary artery ectasia and peripheral varicose veins 19. Nitric oxide has vasodilator effect in the circulatory system and prevents of endothelial dysfunction 20. Indeed, NO is seemed to have an important role in the pathophysiology of varicocele and cardiovascular diseases^21^. Inflammation causes a depletion in circulating NO levels. Association between varicocele and vascular diseases and systemic inflammatory conditions has been reported^22^. Although, EFT was found to be reduced in some conditions having underlying inflammatory mechanisms in their pathogenesis like RVSD, metabolic syndrome and nonischemic dilated cardiomyopathy 12,10, it was found to be increased in others like CAD, IBD and diabetic nephropathy^9^,^11^,^23^. Furthermore, there are conflicting results for insulin resistance, hypertension and dyslipidemia regarding EFT measures^24^–^26^.

We have found that EFT was decreased in high grade varicocele patients compared to healthy controls. Inflammatory mechanisms may play a role in this finding. Decreased EFT size may be associated with varicocele since production of anti-inflammatory adipokines could be decreased in smaller EFT. Since these adipokines have reducing effects on oxidative stress and reactive oxygen species (ROS), decreased secretion of these cytokines may contribute to increased vascular pressures which eventually cause varicose veins 27. Indeed, highgrade varicocele has been found to be associated with ROS and increased oxidative stress 27. Lack of correlation between varicocele and BMI but a negative correlation between EFT and varicocele may suggest that the main production site of regulatory adipokines may be in epicardial fat. However, larger and prospective studies are needed to confirm our results.

There are some limitations of the present study. Some studies in the literature proved that fat tissue secretes both inflammatory and anti-inflammatory adipokines. Since the EFT of varicocele patients was lower than the controls, we shall speculate that the amount of secreted anti-inflammatory adipokines could be reduced compared to controls. Indeed, lack of studying serum levels of these adipokines is a limitation of the present study. Another limitation is the small study population. In addition, cardiac magnetic resonance is a gold standard for visualization of EFT, however, we evaluated only echocardiographic imaging.

## Conclusion

We conclude that high-grade varicocele may be associated with decreased echocardiographic EFT but not with aortic stiffness and FMD. As a practical, cost effective and valuable method, echocardiographic measurement of EFT may provide crucial information about the development of varicocele and measuring it in selected populations may be beneficial.
